# Suppression of Chemotaxis by SSeCKS via Scaffolding of Phosphoinositol Phosphates and the Recruitment of the Cdc42 GEF, Frabin, to the Leading Edge

**DOI:** 10.1371/journal.pone.0111534

**Published:** 2014-10-30

**Authors:** Hyun-Kyung Ko, Li-wu Guo, Bing Su, Lingqiu Gao, Irwin H. Gelman

**Affiliations:** 1 Department of Cancer Genetics, Roswell Park Cancer Institute, Buffalo, New York, United States of America; 2 Div. of Genetic & Reproductive Toxicology, National Center for Toxicological Research, Jefferson, Arkansas, United States of America; Rutgers University, United States of America

## Abstract

Chemotaxis is controlled by interactions between receptors, Rho-family GTPases, phosphatidylinositol 3-kinases, and cytoskeleton remodeling proteins. We investigated how the metastasis suppressor, SSeCKS, attenuates chemotaxis. Chemotaxis activity inversely correlated with SSeCKS levels in mouse embryo fibroblasts (MEF), DU145 and MDA-MB-231 cancer cells. SSeCKS loss induced chemotactic velocity and linear directionality, correlating with replacement of leading edge lamellipodia with fascin-enriched filopodia-like extensions, the formation of thickened longitudinal F-actin stress fibers reaching to filopodial tips, relative enrichments at the leading edge of phosphatidylinositol (3,4,5)P3 (PIP3), Akt, PKC-ζ, Cdc42-GTP and active Src (Src^poY416^), and a loss of Rac1. Leading edge lamellipodia and chemotaxis inhibition in SSeCKS-null MEF could be restored by full-length SSeCKS or SSeCKS deleted of its Src-binding domain (ΔSrc), but not by SSeCKS deleted of its three MARCKS (myristylated alanine-rich C kinase substrate) polybasic domains (ΔPBD), which bind PIP2 and PIP3. The enrichment of activated Cdc42 in SSeCKS-null leading edge filopodia correlated with recruitment of the Cdc42-specific guanine nucleotide exchange factor, Frabin, likely recruited via multiple PIP2/3-binding domains. Frabin knockdown in SSeCKS-null MEF restores leading edge lamellipodia and chemotaxis inhibition. However, SSeCKS failed to co-immunoprecipitate with Rac1, Cdc42 or Frabin. Consistent with the notion that chemotaxis is controlled by SSeCKS-PIP (vs. -Src) scaffolding activity, constitutively-active phosphatidylinositol 3-kinase could override the ability of the Src inhibitor, SKI-606, to suppress chemotaxis and filopodial enrichment of Frabin in SSeCKS-null MEF. Our data suggest a role for SSeCKS in controlling Rac1 vs. Cdc42-induced cellular dynamics at the leading chemotactic edge through the scaffolding of phospholipids and signal mediators, and through the reorganization of the actin cytoskeleton controlling directional movement.

## Introduction

Cell migration is essential for many biological processes such as embryonic morphogenesis, immune responses and wound healing [1]. Indeed, the ability of metastatic tumor cells to disseminate to secondary, distal sites requires the appropriation of signaling pathways that control actin cytoskeletal remodeling, cell polarity, directional motility, cell-cell and cell-extracellular matrix (ECM) adhesion mechanisms, and chemotaxis [1], [2]. These processes are controlled spatiotemporally by physical and functional interactions between cell surface chemoattractant receptors [3], [4], Rho-family GTPases [5], a host of GTPase-regulatory proteins such as guanine nucleotide activating proteins (GAP), guanine nucleotide exchange factors (GEF), and guanosine nucleotide dissociation inhibitors [6], phosphatidylinositol 3-kinases (PI3K) that generate so-called second-messenger phosphatidylinositol(3,4,5)P_3_ (PIP3) from various PIP2 phospholipids [7], and proteins that remodel major actin cytoskeletal structures such as F-actin stress fibers and focal adhesion plaques [4], [8]. There is growing appreciation that the role played in cancer progression by these pathways and mediators makes them attractive targets for therapeutic development [9], [10].

Chemotactic cells typically exhibit directional polarity towards a chemoattractant gradient, with the formation of structures such as leading and lagging domains. In most motile cells, the leading edge is characterized by a fan-like lamellipodium lacking mature focal adhesion plaques and large F-actin stress fibers, with periodic filopodia protrusions seemingly pushed out by F-actin bundles [2]. Activation of the Rho GTPase family members, Rac1 or Cdc42, directs lamellipodia and filopodia formation, respectively [11]. The predominance of Rac-directed lamellipodia formation at leading edges likely relates to increased generation and enrichment of PIP3, due to the activation of chemoattractant receptors that increase local PI3K activity levels [12]. This, in turn, recruits increased levels of Rac-activating proteins via PIP2/PIP3-binding domains such as pleckstrin-homology (PH) and Fab1p-YOPB-Vps27p-EEA1 (FYVE) domains [7]. Additionally, cytoskeletal and signaling pathways activated through increased epithelial-to-mesenchyme transition play a role in potentiating Rac-dependent lamellipodia formation, chemotaxis and invasive potential in cancer cells [13]. Cancer cells also display increased motility rates and directionality [14], relating to cytoskeletal remodeling pathways controlled by an activated axis involving Src-family tyrosine kinases, the focal adhesion kinase (FAK) and PI3K [15], [16].

There is growing appreciation for the role played by so-called “scaffolding proteins” in complex processes such as chemotaxis, through their ability to coordinate signaling and cytoskeletal proteins in a spatiotemporal manner [17], [18]. Characteristics that define scaffolding proteins include multiple, independent protein binding domains, the ability to multimerize (and thereby, amplify signal mediation), and the ability to translocate between cellular domains or compartments (thereby partnering scaffolded signaling enzymes with appropriate substrates).

SSeCKS (Src-Suppressed C Kinase Substrate), the rodent ortholog of human A Kinase Anchoring Protein (AKAP)-12 (or Gravin), is a metastasis suppressor that attenuates oncogenic signaling and motility pathways through its multiple scaffolding domains for signaling mediators such as protein kinase (PK) C and PKA, cyclins, Src and calmodulin [19]. In addition to an F-actin binding domain involved with association with the actin cytoskeleton, SSeCKS also encodes three so-called MARCKS-like PBD known to bind various phosphoinositol phosphates (PIP)[20], which, in addition to the N-terminal myristylation of the αSSeCKS isoform [21], facilitates plasma membrane association [22]. The downregulation of SSeCKS expression is associated with cancer malignancy parameters such as metastasis and recurrence [23], and indeed, SSeCKS is downregulated by oncogenes known to be especially activated in cancer malignancy such as Src, Ras and Myc [24]. In addition to suppressing metastasis by inhibiting tumor-specific expression of vascular endothelial growth factor (VEGF)[25], SSeCKS inhibits parameters of oncogenic motility, such as invasiveness and chemotaxis [26], by re-establishing normalized cytoskeletal architecture including stress fiber and focal adhesion plaque formation. Conversely, SSeCKS re-expression enhances cell adhesion to ECM, correlating with increased transient levels of FAK^poY397^ [27]. Recent evidence indicates that SSeCKS suppresses oncogenic cell motility by directly scaffolding Src and sequestering pools from integrin/growth factor receptor/FAK-enriched plasma membrane domains to lipid rafts, thereby disengaging Src from adhesion- or growth factor-induced activation of MEK-ERK pathways that control pro-oncogenic actin-based cytoskeletal reorganization and podosome formation [27].

In the current study, we addressed the role of SSeCKS in controlling the cellular structures and Rho family GTPases involved in chemotaxis. Chemotaxis by mouse embryo fibroblasts (MEF) or human cancer cell lines, DU145 or MDA-MB-231, could be enhanced by SSeCKS knockdown or genetic loss, or suppressed by forced SSeCKS expression. SSeCKS-deficient cells exhibited increased directional motility towards chemoattractants, marked by thickened, longitudinal F-actin stress fibers ending in predominantly filopodial protrusions at the leading edge. Additionally, the leading edges of SSeCKS-deficient cells showed enrichments of PIP3, activated Cdc42 and Src, and the Cdc42-specific GEF, Frabin. The ability of SSeCKS to rescue formation of leading edge lamellipodia and to suppress chemotaxis required the three PBD, which we show here bind PIP2 and PIP3. Although Src activity was required for chemotaxis, SSeCKS’ ability to suppress chemotaxis did not require its Src scaffolding activity. Taken together, our data strongly suggest that SSeCKS suppresses chemotaxis by differentially organizing PIP3, Rho-family GTPases and their regulatory proteins, Src and regulators of actin cytoskeletal remodeling at the leading edge.

## Materials and Methods

### Cell culture

C57BL/6 wild type (WT) and SSeCKS−/− (KO) primary mouse MEF were derived from E13.5 embryos as described previously [28]. Production of the MEF was performed under protocol 963 M approved by the Institutional Animal Care and Use Committee of Roswell Park Cancer Institute. Pregnant females were euthanized by sodium pentobarbital overdose, and to minimize suffering, embryos were placed in ice-cold PBS followed by decapitation. Cells were maintained in Dulbecco’s modified Eagle medium (DMEM) (Invitrogen/Life Technologies, Grand Island, NY) supplemented with 10% heat-inactivated fetal bovine serum (FBS; Invitrogen), 0.1 mM non-essential amino acids, 2 mM L-glutamine (Mediatech/Corning, Manassas, VA), 0.05 mM 2-mercaptoethanol (Life Technologies) with initial passages plated on gelatinized T25 flasks (0.2% gelatin solution, filter sterilized). Human cancer cell lines, DU145 (prostate; ATCC-CRL-2698) or MDA-MB231 (breast; ATCC-HTB-26), were cultured in RPMI 1640 or DMEM, respectively, containing 10% FBS.

### Antibodies and Reagents

The primary antibodies (Ab) used include rabbit polyclonal Abs: SSeCKS [21], GAPDH (sc-25778, Santa Cruz Biotechnology, Santa Cruz, CA), PKC-ζ (sc-216, Santa Cruz), Akt (#9272, Cell Signaling, Beverly, MA); mouse monoclonal Abs: His_6_ (G020, Abm, Richmond, BC, Canada), glutathione S-transferase (GST; Abm, G018), Rac1 (ARC03, Cytoskeleton, Denver, CO) Cdc42 (sc-8401, Santa Cruz), PIP3 (Z-P345, Echelon, Salt Lake City, UT), PIP2 (Z-P045, Echelon), Frabin (sc41718, Santa Cruz); goat polyclonal Ab: Par6 (sc-14403, Santa Cruz). The following secondary Abs were purchased from Invitrogen: anti-rabbit Alexa Fluor 568 (A11011) and Alexa Fluor 488 (A11008), anti-mouse Alexa Fluor 568 (A11031) and Alexa Fluor 488 (A11001). Rhodamine-labeled phalloidin and fluorescein isothiocyanate (FITC)-conjugated phalloidin were from Invitrogen. Human AKAP12-si-RNA (sc-40305) and mouse Fgd4 siRNA (siGENOME upgrade siRNA, D-055412-03) were from Santa Cruz and Thermo Scientific (Waltham, MA), respectively. Other reagents include LY294002 (Cell Signaling), SKI-606 (Selleckchem, Houston, TX), digitonin (D141, Sigma, St. Louis, MO), Human epidermal growth factor (EGF) (PHG0311, Invitrogen), LipoD293 (SignaGen, Rockville, MD), Lipofectamine 2000 and Oligofectamine (Invitrogen).

### Plasmids and transfection

pcDNA3.1-SSeCKS was produced by transferring the 5.8 Kb EcoRI fragment from pBluescript–SSeCKS [21]. The Δ2–552 deletions were produced in pcDNA3.1-SSeCKS and SSeCKS-GFP (green fluorescent protein) [29] by a long range inverse PCR technique as described previously [30] using the SSeCKS#2 and #552 primer sets containing a unique Cla I site (underlined) and an Asu II site (bold):

SSeCKS#2F:^5′^ATCGATCGATCATGGCTCAGTGGCTCTTCTACTCCCGC^3′^; SSeCKS#2R:^5′^ATCG**TTCGAA**CATGTGTC.

GACCTCGAGATCTGAGTCCG^3′^; SSeCKS#552F:^5′^ATCGATCGATTCTGCGTCGTCCCCCGAGGAGCCTGAGG^3′^; SSeCKS#552R:^5′^ATCG**TTCGAA**TCTGCGTCGTCCCCCGAGGAGCCTGAGG^3′.^

GST-SSeCKS fusion constructs were described previously [29]. PH-Akt-GFP, His-WASP-CBD (Cdc42 binding domain), GST-PAK-PBD, CA-PI3K (CA-p110) were gifts of Henry R. Bourne (University of California San Francisco), K. Hahn (Univ. North Carolina), A. Bakin (RPCI) and J. Downward (London Res. Inst.), respectively. Cells (3×10^5^/well in 6-well dishes) were transiently transfected using LipoD293 and 5 µg plasmid DNA according to the manufacturer’s instructions, and lysates were produced using radioimmunoprecipitation assay (RIPA) buffer [21] 18–40 h later. siRNA transfections were carried out in 6-well dishes using 20–50 nM siRNA in Lipofectamine 2000 or Oligofectamine according to manufacturer’s instructions. Lysates were prepared 48–72 h later.

### Chemotaxis assays

#### Boyden chamber

Chemotaxis assays were performed in 24-well modified Boyden chambers as described previously [31]. The chemoattractants (10% FBS or 20 ng/ml of platelet-derived growth factor-BB [PDGF-BB]) were loaded into the lower chamber containing 600 µl of serum-free DMEM. MEF (5×10^4^ cells/ml), DU145 or MDA-MB-231 cells (10^5^ cells/ml) in 200 µl of serum-free DMEM were then added atop polyethylene terephthalate membrane inserts (8 µm pores) in the upper chambers of the transwell apparatus (BD Bioscience, San Jose, CA). After incubation for 3 h (MEF) or 16 h (DU145 and MDA-MB-231) at 37°C in a 5% CO_2_ incubator, cells on the top of the insert were removed by wiping with a cotton swab. Migrating cells on the inserts were fixed and stained using Diff-Quik Stain Set (Dade Behring Inc, Newark, DE), and analyzed by phase-contrast microscopy (Nikon Eclipse TS100 Phase Contrast Inverted Microscope, Nikon USA, Melville, NY) by counting at least five visual fields containing at least 10 cells/field, and variations were calculated as standard error (S.E.). Relative chemotaxis is defined as the mean level for each control condition (e.g.- WT MEF, control siRNA or vector alone) set at a value = 1.

#### Agarose spot assay

Chemotaxis to attractants in agarose spots was performed as described by Wiggins and Rappoport [32]. Briefly, a sterile 0.5% low-melting point agarose (Ultrapure LMP agarose; Invitrogen) in PBS solution was boiled, cooled to 40 °C, and 90 µl was added to 10 µl of chemoattractant (7 µl PBS plus 3 µl of EGF/PDGF stock (1 mg/ml each)). 10 µl spots were placed on sterile 22 mm^2^ coverslips in 6-well dishes and allowed to solidify at 4°C. Cells were then plated onto these coverslips, allowed to adhere for 3–4 h, whereupon the media was replaced with DMEM/0.5% FBS. Control agarose spots contained PBS alone.

#### Directional motility

During chemotaxis in the agarose-spot system, cell movements toward agarose spot containing EGF/PDGF were monitored by phase contrast microscopy using a 40x objective lens (T1-SNCP:Nikon). Images were collected every 20 min for 16 h, and velocity and directionality were determined by tracking the positions of at least 20 individual cells/condition. Velocity was calculated by measuring displacement from start to end point and dividing by migration time. Chemotactic motility was evaluated statistically using a forward migration index (FMI)[33], as the linear distance from starting to ending point a cell moved if it had moved directly towards the chemoattractant gradient (h), divided by “b”, the direct vector from the cell’s start (time-point 1) to end (time-point 5).

### Wound scratching assay

Cell motility by MEF into a monolayer wound was performed as described previously [31] in triplicate over a total of 24 h.

### Immunofluorescence analysis (IFA)

MEF cells grown on the glass coverslips (22 mm^2^) were washed with phosphate-buffered saline (PBS) and fixed for 15 minutes in 4% paraformaldehyde in PBS, pH 7.5, at room temperature (RT) followed by permeabilization with 0.05% Triton X-100 in PBS for 10 minutes. For PIP2 and PIP3 staining, cells were permeabilized with digitonin (10 µg/ml) for 10 min at RT. Following three washes with PBS, cells were blocked with PBS containing 3% BSA or 5% FBS for 1 h, and then incubated in primary Abs (1∶25–100 in blocking buffer) for either 1–2 h at RT or overnight at 4°C. Where indicated, cells were fixed with ice-cold 60% acetone, 3.7% paraformaldehyde in PBS for 20 minutes at −20°C. In order to assess Rac1-GTP and Cdc42-GTP staining, fixed cells were pre-incubated with 3% BSA for 1 h at RT and incubated overnight at 4°C with 50 µg GST-PAK-PBD (specific for both activated-Rac and -Cdc42) or His-WASP-CBD (specific for activated-Cdc42). Following three PBS washes (10 min each at RT), the cells were incubated overnight at 4°C with either mAb anti-GST or mAb anti-His and washed three times with PBS. Incubation with secondary Abs (1∶1000 in blocking buffer) for 1 h at RT included either goat anti-mouse IgG conjugated to Alexa Fluor 568 or Alexa Fluor 488, or goat anti-rabbit IgG conjugated to Alexa Fluor 568 or Alexa Fluor 488. F-actin filaments were stained with rhodamine- or FITC-conjugated phalloidin, and nuclei were stained with 4′,6-diamidino-2-phenylindole. Following staining, cells were washed with PBS and then sealed under coverslips with a drop of Prolong anti-fade reagent (Invitrogen). The fluorescent images were acquired on an inverted microscope (Nikon TE 2000) using a 60x oil immersion objective. Digital images were obtained using MetaMorph software (Molecular Devices, Sunnyvale, CA). For quantitative measurements of fluorescence at the leading edges of cells, a cell’s chemotactic leading edge (a 0.5 µm mask into the cell starting from periphery) was traced and the average fluorescence intensity (in pixels) was measured using ImageJ software (http://rsbweb.nih.gov/ij/). Relative fluorescence intensity was provided as the ratio of leading edge signal to whole cell signal in at least five different cells from each of three independent experiments.

### Immunoblotting (IB) analysis

RIPA cell lysates containing freshly added inhibitors (1 mM phenylmethylsulfonyl fluoride, 10 mM NaF, 1 mM Na_3_VO_4_) and Complete Protease Inhibitor Mixture (Roche Diagnostics) were analyzed by IB as described previously [34], using secondary anti-rabbit IgG Alexa 680 Ab followed by visualization using an Odyssey Infrared Imaging System (LI-COR Biosciences, Lincoln, NE).

### Purification of GST/His-fusion proteins

The growth of BL21-pLysS bacteria, induction of GST- or His-tagged fusion proteins with isopropyl β-D-thiogalactopyranoside (1 mM) and protein isolation have been described previously [35].

### Rac and Cdc42 activation assays

Identification of GTP-bound Rac or Cdc42 proteins from lysates using GST-PAK-PBD-beads was described previously [35].

### Protein-lipid binding assays

Hydrophobic membrane strips containing eight phosphoinositides and seven related lipids (PIP-Strips, Echelon) were blocked with 3% BSA in PBS for 1 h at RT. The strip was then incubated for 1 h in blocking buffer supplemented with 1 µg/ml GST or GST-SSeCKS protein. After washing thrice (5 min each) with PBS-T (0.1% v/v Tween-20), the strips were incubated for 1 h at room temperature with mAb anti-GST Ab (1∶1000 in blocking buffer), washed thrice with PBS-T, incubated (1 h at RT) with horseradish peroxidase-conjugated anti-mouse Ig (1∶5000 in blocking buffer), washed thrice with PBS, incubated with enhanced chemiluminescence reagent (ECL kit, Roche Applied Science) and autoradiographed. For the PIP-bead binding assay, 5 µg of GST or GST-SSeCKS proteins were incubated (on a rotator) with 25 µl of PIP beads (Echelon) at RT for 1 h in blocking buffer, washed thrice with PBS-T and then the beads were analyzed by IB for GST.

### Statistical analyses

All experiments were repeated at least thrice and then analyzed by un-paired *t* test and Tukey’s method for statistical significance using Prism v.3 (GraphPad Software, La Jolla, CA), with data shown as mean ± S.E.

## Results

### Loss of SSeCKS leads to enhanced chemotaxis accompanying morphological changes at the leading edge

**Figure 1:**
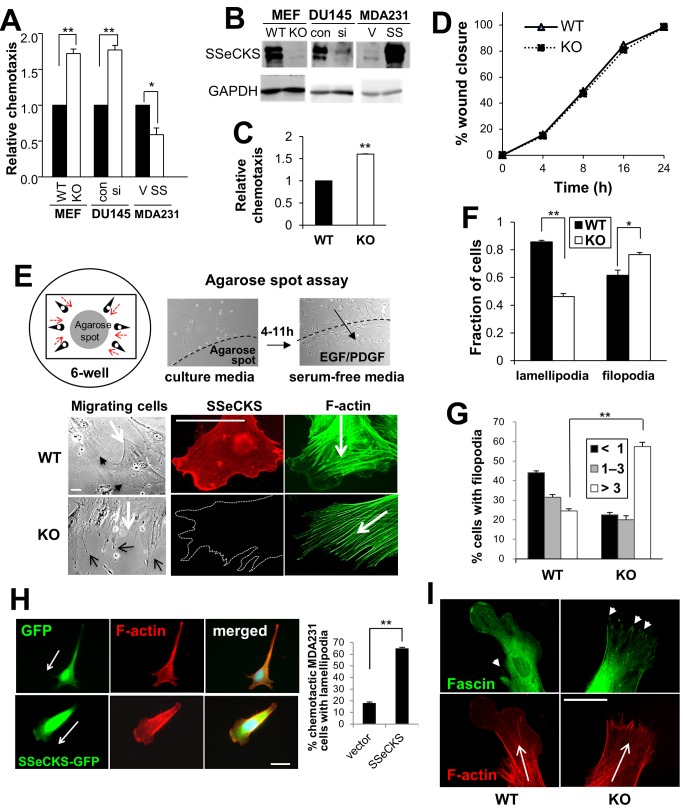
SSeCKS inhibits chemotaxis and affects leading edge protrusions. *A*, Relative chemotaxis of MEF (WT or KO), DU145 (transfected with control [con] or human SSeCKS-siRNA [si]) and MDA-MB-231 cells (transfected with empty vector [V] or an SSeCKS-GFP expression plasmid [SS]), as measured in Boyden chamber assays using serum as the chemoattractant. *Error bars*, S.E. of triplicate assays. *, p<0.02, **, p<0.005. *B*, IB analysis of SSeCKS and GAPDH levels in the cells described in Panel A. *C*, Relative chemotaxis of WT or KO MEF using PDGF as the chemoattractant in Boyden chamber assays. *Error bars*, S.E. of three wounding fields in two independent experiments. **, p<0.02. *D,* Relative ability of WT or KO MEF to close wound scratches, based on measuring three wound field gaps at a given time in triplicate experiments. *E*, Agarose chemotaxis spot assay. *Top: left panel-* cartoon of motile cells (*black*) moving towards (*red arrows*) an agarose spot containing chemoattractant; *right panels-* example of assay without (“culture media” plus PBS; *left*) or with chemoattractant gradient (“serum-free media” plus EGF/PDGF in spot; *right*). *Bottom:* Leading edges of chemotactic WT cells predominantly display lamellipodia whereas those of KO cells predominantly display filopodia-like extensions. *Left panels-* phase contrast microscopy of chemotactic cells. *Open-head black arrows*, filopodia; *closed-head black arrows*, lamellipodia; *white arrows*, chemotaxis direction. *Middle and right panels-* IFA staining of SSeCKS or F-actin in WT or KO MEF. *Arrows*, chemotaxis direction. *Scale bar*, 10 µm. *F*, Fraction of chemotactic WT or KO cells with leading edge lamellipodia or filopodia. *Error bars*, S.E. of 5 visual fields with at least 10 cells/field in three independent experiments. *, p<0.02, **, p<0.005. *G*, Percentage of chemotactic WT or KO cells in Panel F with <1, 1–3 or >3 filopodia/leading edge. **, p<0.005. *H,* Induction of lamellipodia formation in MDA-MB-231 cells transfected with SSeCKS-GFP (*vs. GFP vector alone*), as shown by IFA for GFP (*left panels*) or F-actin (*center*), or following quantification (*graph, right*). *Arrows*, chemotaxis direction. *Scale bar*, 10 µm. *Error bars*, S.E. of 5 visual fields with at least 10 cells/field in three independent experiments. *I*, IFA for fascin and F-actin in WT and KO MEF. *Short arrows*, fascin-staining filopodia. *Long arrows,* chemotaxis direction.

Our group reported previously that SSeCKS suppressed chemotaxis in rat MATLyLu prostate cancer cells without affecting cell motility in monolayer wounding assays [26]. Because chemotaxis assays typically measure parameters of individual cell migration whereas monolayer wounding reflects collective movements of cell sheets, this suggests that SSeCKS controls specific aspects of individual cell movement. To further address the role of SSeCKS in controlling chemotaxis, we compared motility of cells in Boyden chambers assays consisting of chemoattractants in the bottom chamber. SSeCKS-null (KO)-MEF displayed increased chemotaxis relative to matched WT MEF against serum (Fig. 1A) or PDGF-BB (20 ng/ml) (Fig. 1C). SSeCKS levels in DU145 and MDA-MB-231 are considered downregulated compared to untransformed epithelial cells [36], although to a greater extent in MDA-MB-231 cells, and thus, we sought to knockdown SSeCKS/AKAP12 levels in DU145 and overexpress SSeCKS in MDA-MB-231 cells. The siRNA-mediated knockdown of human SSeCKS/AKAP12 in DU145 prostate cancer cells resulted in increased chemotaxis to serum, whereas its overexpression in MDA-MB-231 breast cancer cells inhibited chemotaxis (Fig. 1A). The knockdown of both endogenous α and β SSeCKS isoforms (upper and lower bands, respectively; ref. [37]) or overexpression of ectopic αSSeCKS isoform in MDA-MB-231 cells was verified by immunoblotting (Fig. 1B). Significantly, the loss of SSeCKS had no effect on cell migration involving the closure of monolayer wounds (Fig. 1D). Taken together, these data suggest that SSeCKS regulates specialized motility such as chemotaxis in both untransformed and cancer cells.

Chemotactic cells are marked by polarized actin cytoskeleton-based structures, such as lamellipodia, filopodia, blebs, and invadopodia at the so-called leading edge [2]. Filopodial formations, produced by activated Cdc42, are responsible for chemosensing in neuronal growth cone guidance during axonal extension and in one report [38], transendothelial chemotaxis by mesenchymal stem cells. In contrast, lamellipodial formations, produced by activated Rac1, generally initiate and facilitate directional cell migration towards the gradient of chemoattractants. Given findings that SSeCKS is a membrane- and cytoskeleton-associated protein [19] that regulates actin polymerization [39], we addressed whether its ability to regulate chemotaxis correlates with a role for controlling leading-edge dynamics. Thus, we compared the morphologies of WT or KO MEF migrating toward chemoattractants embedded in an agarose spot as described in Fig. 1E. In this assay, cells are seeded in serum-free media onto surfaces already containing spots with either chemoattractant or PBS. Whereas chemotactic WT MEF predominantly displayed large lamellipodia at their leading edges (Fig. 1E, *bottom,* closed head arrows; Fig. 1F), KO MEF predominantly displayed elongated, filopodia-like protrusions (Fig. 1E, *bottom,* open head arrows; Fig. 1F). KO MEF also exhibited increased numbers of filopodia/cell compared to WT controls (Fig. 1G). Indeed, the forced re-expression of SSeCKS-GFP in MDA-MB-231 human breast cancer cells increased the frequency of lamellipodia at leading edges compared to cells transfected with vector alone (Fig. 1H).

In order to further characterize the leading edge extensions in KO MEF, WT and KO cells were stained for fascin, an actin-bundling protein enriched in filopodia [40] whose expression is associated with metastatic invasiveness in cancer cells [41]. The enrichment of fascin staining at the F-actin-rich tips of the KO MEF projections confirms their identify as filopodia (Fig. 1I). We also observed that F-actin stress fibers, identified by phalloidin staining, typically did not penetrate from the cell center into the lamellipodial leading edge in WT MEF, yet they extended to the ends of filopodial protrusions in leading edges of KO MEF (Figs. 1E&I). This was also marked by increased stress fiber thickening and longitudinal orientation in KO vs. WT MEF, a characteristic we previously described following SSeCKS knockdown in renal stellate mesangial cells [42].

### SSeCKS regulates directional motility

**Figure 2:**
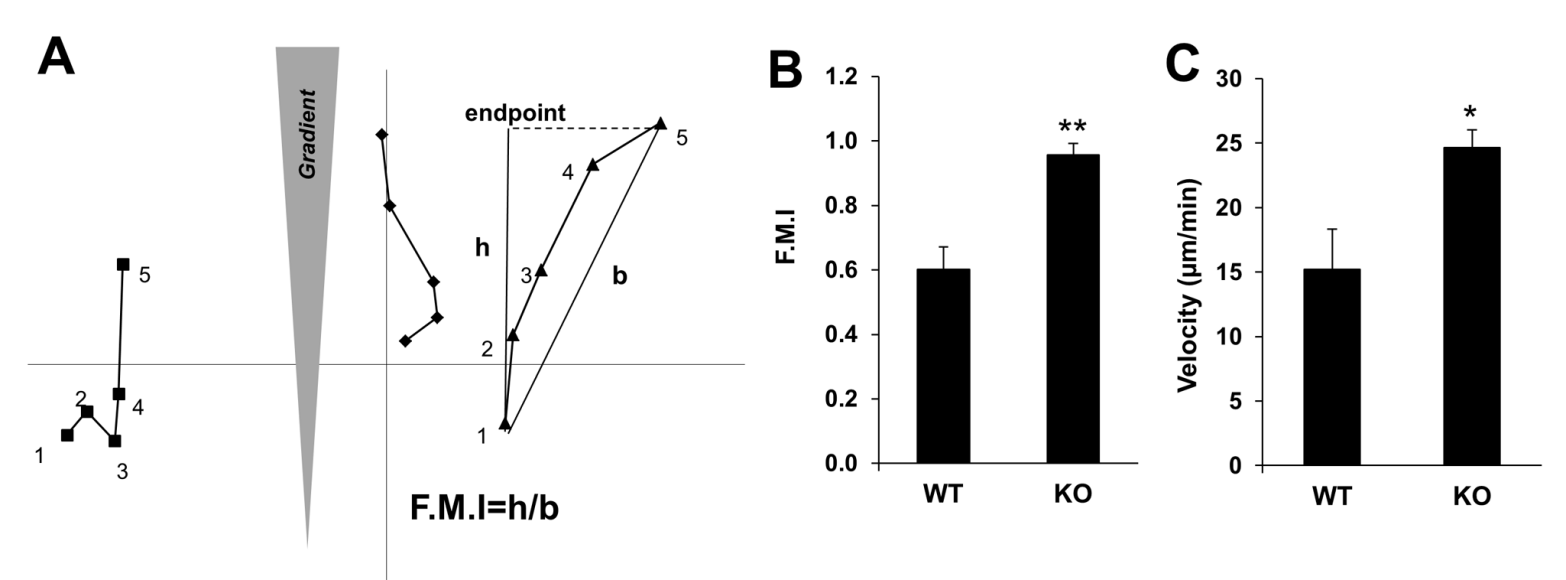
SSeCKS inhibits the rate and directionality of chemotactic cells. *A*, Hypothetical chemotactic paths of three cells (square, diamond or triangle) based on five time measurements (numbered). The forward migration index (FMI) is calculated as the distance “h”, if a cell theoretically travelled directly towards the chemoattractant source over time-points 1 to 5, divided by “b”, the direct vector from the cell’s start (time-point 1) to end (time-point 5). A cell moving in a straight, direct line towards a chemoattractant would have an FMI = 1. KO MEF have increased FMI (*B*) and velocity (*C*) compared to WT MEF. *, p<0.05, **, p<0.01 for at least 20 cells/time-point/condition.

Chemotaxis efficiency is affected by both the directionality and velocity of cell migration, parameters associated with the number, orientation and type of protrusions at the leading edge, as well as their interaction with dynamic changes in the remodeling of the actin cytoskeleton [14]. We addressed whether SSeCKS could regulate chemotaxis through control of migration directionality or velocity, given our results that it affects the dynamics of F-actin stress fiber and leading-edge protrusion assembly (Fig. 1). Cell movement of WT or KO MEF towards chemoattractant-laden agarose spots was measured over time, where directionality was calculated by a forward motion index (FMI) based on the distance traveled by a cell (origin to endpoint) when vectored directly toward the agarose spot (h), divided by the vector distance from the actual origin to endpoint (b) (Fig. 2A). Our data indicate that KO MEF have roughly 1.5-fold greater directionality than WT controls, and a small, but significant increase in velocity (Fig. 2B). These data suggest that SSeCKS controls chemotaxis by attenuating chemoattractant sensing and/or cytoskeletal reorganization mechanisms.

### SSeCKS regulates chemotaxis through association of it polybasic domains with phosphoinositol phosphates

**Figure 3:**
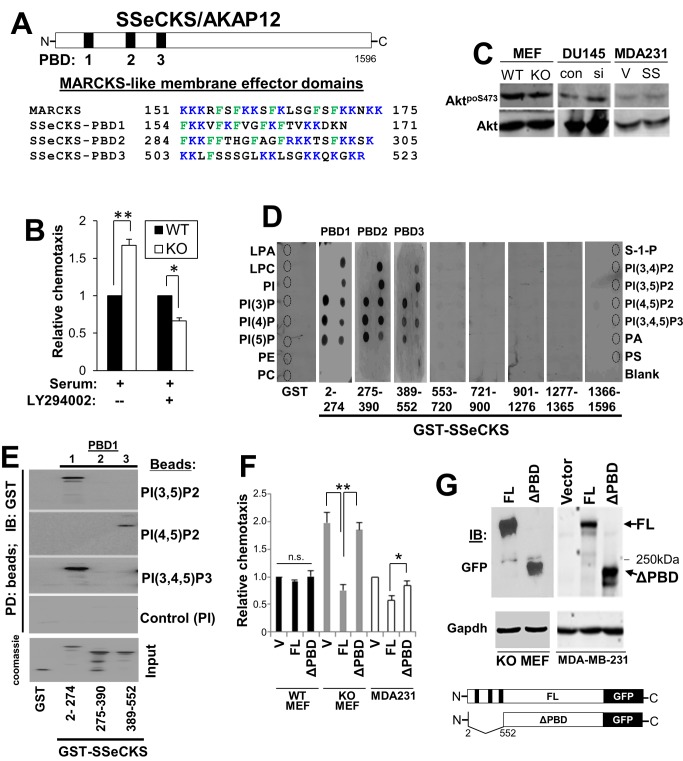
SSeCKS encodes three PBD that are required for inhibition of chemotaxis. *A*, Location (*top*) and sequence (*bottom*; flanking residue numbers) of the SSeCKS PBD compared to the MARCKS membrane effector domain. *B*, WT or KO MEF were assessed for chemotaxis towards serum in the presence or absence of the PI3K inhibitor, LY29004. *Error bars*, S.E. of two independent experimental duplicates. *, p<0.02, **, p<0.01. *C,* IB analyses for Akt^poS473^ or total Akt in lysates of cells from Fig. 1B. *D,* Overlay assay of PIP-Strips with GST or GST-fusion proteins. Phospholipid spots (*identified as broken-lined circles*) are labeled on the left and right sides. Production of the GST and GST-SSeCKS fusion proteins is described in Guo et al. [29]. *E*, PIP-bead binding assay. PIP-beads binding to GST or GST-SSeCKS proteins (aliquots shown on a Coomassie-stained gel, *bottom*) were assessed by SDS-PAGE by IB for GST. *F,* FL SSeCKS, but not ΔPBD-SSeCKS, rescues chemotaxis inhibition (*left panel*) in KO MEF and MDA-MB-231, but not in WT MEF, compared to vector (V) alone. *, p<0.05, **, p<0.005. *G,* GFP-IB of full-length (FL) and ΔPBD SSeCKS-GFP proteins (*arrows*) expressed in KO MEF or MDA-MB-231 cells, compared to GADPH-IB as a loading control. *Bottom-* cartoon of FL- vs. ΔPBD SSeCKS-GFP constructs in which the 3-PBD (*black* bars) are lost in the a.a. 2–552 deletion. *“250* *kDa”*, marker protein.

The generation of phosphoinositol phosphate lipid second messenger pools, especially PI(4,5)P2 and PI(3,4,5)P3, by various phosphoinositide kinases [43], [44] whose activities localize at the leading edge, are known to be crucial steps for controlling cell motility [7]. For example, the Rho GTPase family members, Rac and Cdc42, which promote lamellipodia and filopodia formation, respectively [45], can be activated by multiple GEFs. GEFs are brought to plasma membrane activation sites via their phosphoinositide-binding domains, including PH, phox homology (PX) and FYVE domains [46]. SSeCKS contains three N-terminal PBD that share sequence and charge homology with the membrane effector domain of MARCKS protein [20], [22], [47] (Fig. 3A). These regions have been implicated in mediating reversible plasma membrane interaction through the binding of phospholipids [48], and in addition to SSeCKS’ N-terminal myristylation [21], are required for SSeCKS’ plasma membrane association [22]. Because the enhanced chemotaxis of KO MEF was sensitive to the PI3K inhibitor, LY294002 (Fig. 3B), yet SSeCKS had no effect on AKT protein or activation levels (Fig. 3C), we hypothesized that SSeCKS regulates chemotaxis through its interactions with phosphoinositides. To address this, we performed PIP-strip overlay assays, in which nitrocellulose membranes spotted with 15 phospholipids were incubated with GST fusions of various SSeCKS domains. After washing, the bound proteins were identified by immunoblotting for GST antigen. GST-SSeCKS fusions containing PBD1 and 2 (a.a. 2–274 and 275–390) showed binding activity to phosphatidic acid (PA) but not to phosphatidylinositol (PI), phosphatidylethanolamine (PE), phosphatidylserine (PS) or phosphatidylcholine (PC) (Fig. 3D). All three PBD bound to the mono-, di- and tri-phosphorylated PI, whereas no binding was detected to lysophosphatidic acid (LPA), lysophosphatidylcholine (LPC) or sphingosine-1-phosphate (S-1-P). All three PBD strongly bound PI(3)P, PI(4)P or PI(5)P, as well as the PIP2 and PIP3 lipids, although PBD3 preferred PI(3,5)P2 and PI(3,4,5)P3 (Fig. 3D). As a negative control, GST alone or GST-SSeCKS fusions not containing PBD failed to bind any of the lipids on the strip. Based on the notion that the MARCKS membrane effector domain binds acidic lipids through its concentration of positively-charged lysine and arginine residues [20], we employed a solution-based binding assay of GST-SSeCKS proteins to PIP-beads. PBD1 bound to beads covalently linked to PI(3,5)P2 and PI(3,4,5)P3 but not to PI(4,5)P2 or PI (control), whereas PBD3 bound to PI(4,5)P2 (Fig. 3E). The difference between the two assays is unclear, however, the PIP-bead assay binding kinetics are shorter, suggesting that the binding conditions for the PIP Strip favor saturation of the GST-SSeCKS fusion proteins. In contrast, the PIP-bead assays suggest that there is greater selectivity for PIP2/PIP3 binding amongst the PBD.

We then addressed whether PBD domains are required for SSeCKS to regulate chemotaxis. WT or KO MEF transiently transfected with full-length (FL) SSeCKS-GFP cDNA or an SSeCKS-GFP mutant deleted of its PBD (“ΔPBD”: deletion of a.a. 2–552; Fig. 3F) were assessed for chemotactic activity by scoring for GFP-positive attracted cells. The enhanced relative chemotaxis of KO MEF was decreased to levels in WT MEF by FL but by not ΔPBD SSeCKS (Fig. 3F). Similar results were demonstrated in MDA-MB-231 cells (Fig. 3F). As controls, we showed that neither FL nor ΔPBD SSeCKS affected WT MEF chemotaxis (Fig. 3F), and that KO MEF or MDA-MB-231 cells expressed similar ectopic levels of the FL and ΔPBD proteins (Fig. 3G). These findings suggest that PBD binding to phospholipids plays a role in SSeCKS’ ability to regulate chemotaxis.

### SSeCKS alters PIP2 and PIP3 enrichment at the chemotactic leading edge

**Figure 4:**
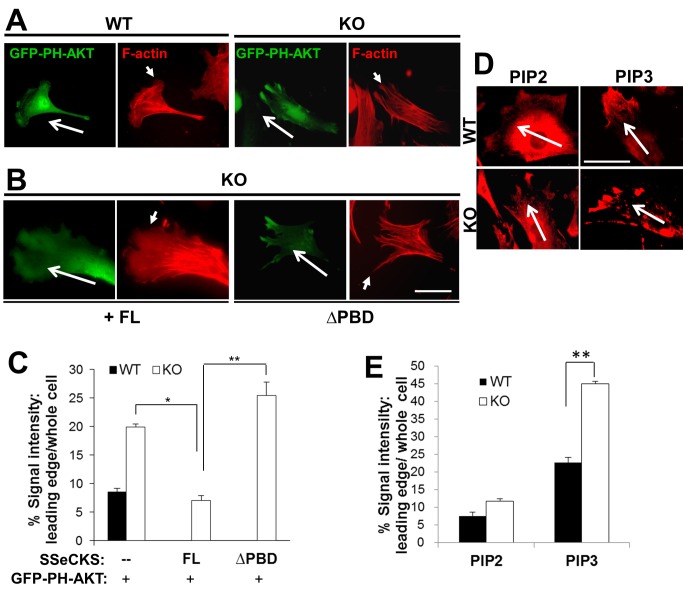
SSeCKS attenuates PIP3 enrichment at the leading edge. *A*, Chemotactic WT or KO MEF (*long arrows*: chemotaxis direction) transiently expressing the PIP3 reporter, GFP-PH-AKT, and stained for F-actin, showing enrichment of PIP3 in the leading edge filopodia of KO cells. *Short arrows*, leading edge lamellipodia (WT cells) or filopodia (KO cells). *B*, Re-expression of FL-, but not ΔPBD-, SSeCKS-GFP rescues leading edge lamellipodia formation in KO MEF and suppresses leading edge enrichment of the GFP-PH-AKT reporter. *Scale bar*, 10 µm. *C*, Quantification of leading edge GFP-PH-AKT levels (normalized to total cell GFP) determined for 3 fields containing at least 10 cells/field in 2 independent experiments. *Error bars*, S.E. *, p<0.01, **, p<0.005. *D*, PIP2 or PIP3 in chemotactic leading edges of WT or KO MEF by IFA or quantified as in *E*. **, p<0.01. *Scale bar*, 10 µm.

Given that SSeCKS functions as a scaffolding protein and that PBD domains are critical for the ability of SSeCKS to inhibit chemotaxis, it is conceivable that SSeCKS suppresses chemotaxis by controlling the enrichment of phosphoinositol phosphates at the leading edge through its scaffolding function. To address this, WT and KO MEF were transfected with a GFP-PH-AKT probe, which preferentially binds PI(3,4,5)P3 [49] and which shows enriched staining in the leading edge lamellipodia of fibroblasts chemotaxing to PDGF [50]. Consistent with their differences in leading edge protrusions, WT MEF showed concentrated GFP-PH-AKT staining at the leading edges of lamellipodia whereas KO MEF showed enrichments in filopodia-like extensions (Fig. 4A). Re-expression of full-length (FL)-SSeCKS in KO MEF restored lamellipodia formation, similar to the effects found in MDA-MB-231 cells (Fig. 1H), as well as the GFP-PH-AKT edge staining pattern found in WT MEF (Fig. 4B). In contrast, ΔPBD-SSeCKS failed to alter the filopodial staining pattern in KO MEF. Interestingly, the pattern of F-actin stress fibers protruding to the leading edge filopodia in KO MEF was altered by FL-SSeCKS overexpression, such that stress fibers ended prior to the leading edge lamellipodia (Fig. 4A). However, ΔPBD-SSeCKS overexpression failed to alter the typical stress fiber formation in KO MEF. The ability of SSeCKS to alter PIP3 levels at the leading edge was assessed by measuring relative GFP-PH-AKT staining intensities 0.5 µm from the leading cell edge as a fraction of total cell GFP-PH-AKT staining, normalized for cell volume. KO MEF exhibited 2.5-fold more PIP3 enrichment at their leading edges compared to levels in WT MEF (Fig. 4C). The finding that FL-SSeCKS, but not ΔPBD-SSeCKS, decreases PIP3 leading edge staining levels to those in WT cells strongly suggests that SSeCKS attenuates chemotaxis by scaffolding PIP3 away from leading edge structures. We also compared the localization of endogenous PIP2/PIP3 at the leading edge of WT and KO MEF by immunofluorescence staining using PIP2- or PIP3-specific antibodies (Fig. 4D). Although WT and KO MEF had similar PIP2 levels in their leading edge protrusions, KO MEF had 2-fold higher relative levels of PIP3 in their leading edge filopodia compared to levels in the WT leading edge lamellipodia (Figs. 4D & E).

### SSeCKS controls the localization of chemotaxis regulators

**Figure 5:**
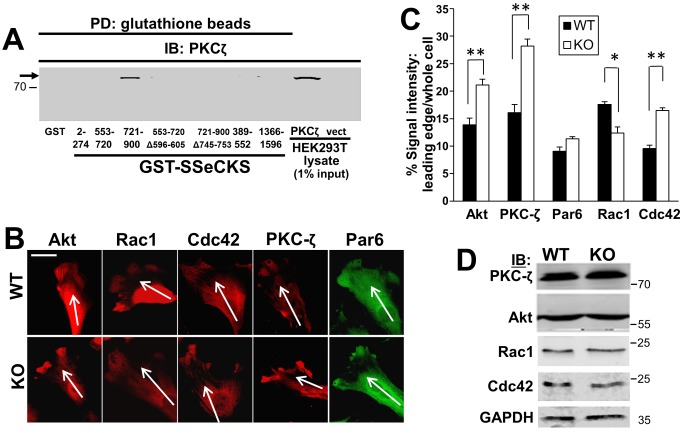
SSeCKS controls the localization of signaling mediators that regulate leading edge protrusion formation. *A,* SSeCKS scaffolds PKCζ. PKCζ IB analysis of HEK293T cell lysates transfected with PKCζ-GFP or pEGFP (vect) after pull down using GST- or GST-SSeCKS-beads. Aliquots of lysates (5 µg, representing 1% input) are shown on the right. Note that PKCζ (*arrow*) binds one of the two PKC-binding domains identified previously [29], and that deletion of the minimal binding domain (a.a.- 745–753) abrogates this binding. *B,* Chemotactic WT or KO MEF were stained by IFA for Akt, Rac1, Cdc42, PKC-ζ or Par6, and the leading edge staining was quantified in *Panel C* as in Fig. 4 *C*. *, p<0.05, **, p<0.01. *Scale bar*, 10 µm. *D*, IB analysis for the proteins stained in panel *A*, plus GAPDH controls. MWt markers are shown at right.

The localized enrichment of PIP2 and PIP3 at the leading edge is known to recruit and activate several signaling mediators of chemotaxis, such as Rac1, Cdc42 and AKT [2]. Additionally, an important mechanism that influences chemotaxis is the ability of activated Cdc42 to recruit Par6 and atypical PKC isoforms to the polarized leading edge [51], [52]. We speculated that SSeCKS might alter chemotaxis by affecting the recruitment of one or more of these factors at the leading edge. Indeed, in the case of PKC, SSeCKS can directly scaffold conventional, novel [29], and atypical (Fig. 5A) isoforms. KO MEF exhibited increased enrichment levels of Akt, PKC-ζ and Cdc42 at leading edge filopodial protrusions, whereas WT MEF exhibited higher enrichment levels of Rac1 in lamellipodia (Figs. 5B & C). In contrast, SSeCKS did not seem to affect Par6 enrichment at the leading edge. Significantly, WT and KO MEF showed no major differences in the total cellular levels of these proteins (Fig. 5D).

### Selective activation of Cdc42 in SSeCKS-null cells

**Figure 6:**
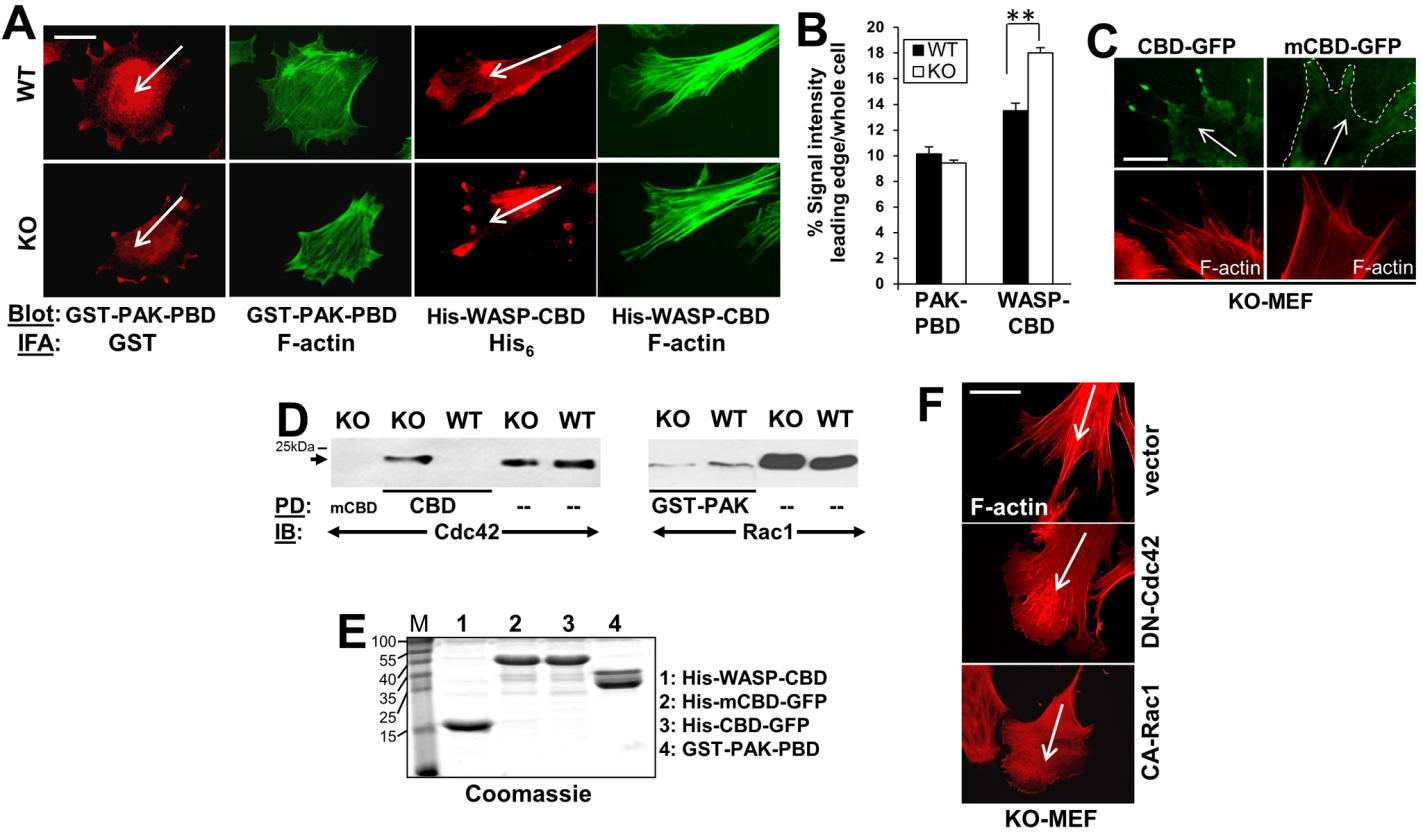
Enriched active Cdc42 in chemotactic leading edges in the absence of SSeCKS. *A*, Chemotactic WT or KO MEF were analyzed for localization of active Rac1 and Cdc42 by overlay assay as described in Experimental Procedures. Cells overlaid with GST PAK-PBD were probed by IFA for GST or F-actin, whereas cells overlaid with His-WASP-CBD were probed for His_6_ or F-actin. *B*, The leading edge staining enrichment for GST-PAK-PBD or His-WASP-CBD was quantified as described in Fig. 4*C*. **, p<0.02. *C*, KO-MEF transfected with CBD-GFP or mCBD-GFP, showing GFP fluorescence (*upper panels*) or F-actin staining (*lower panels*). *D*, Cdc42 activation in KO-MEF pseudopodia. *Left panel-* Pseudopodia isolated from WT- or KO-MEF as described in Materials and Methods were subjected to pulldown (PD) using CBD- or mCBD-beads followed by IB analysis for bound, active Cdc42. IB of direct lysates (“–”) are shown at right. *Right panel-* IB analysis of total MEF lysates (“–”) vs. PD using GST-PAK-PBD beads. *Arrows*, Cdc42 or Rac1 protein. *E,* Coomassie stained gel of the proteins used for PD in panel *D*. *F,* Lamellipodia or filopodia formation in KO MEF expressing DN-Cdc42 or CA-Rac1 (vs. vector). *Scale bar* (for whole figure), 10 µm. *Long white arrows*, chemotaxis direction.

Based on the SSeCKS-regulated morphological differences and subcellular localization patterns of chemotaxis signaling mediators at the leading edge, we focused on the possibility that SSeCKS differentially regulates Rac and Cdc42 activation at leading edge membrane protrusions. The finding that the leading edge protrusions of KO MEF are predominantly filopodia- rather than lamellipodia-like (Fig. 1F) suggests a hyperactivation of Cdc42. To observe localized activated Rac and Cdc42, we overlaid fixed, chemotactic WT or KO MEF with either GST-tagged PAK-PBD, which binds active, GTP-bound forms of both Rac1 and Cdc42 [53], or His-tagged WASP-CBD, which preferentially binds GTP-Cdc42 [54], and the slides were then processed for epitope-tag-specific immunofluorescence staining. Enriched staining for the GST-PAK-PBD probe was found in lamellipodia and filopodia structures of WT and KO MEF (Fig. 6A). However, whereas the leading edge lamellipodia of WT MEF showed a mild enrichment of diffusely staining His-WASP-CBD probe, KO MEF showed strong punctate enrichments at the ends of their filopodial protrusions (Fig. 6A). Quantification of the GST-PAK-PBD and His-WASP-CBD staining within 0.5 µm of the leading edge confirmed a significant increase in His-WASP-CBD enrichment in KO vs. WT cells (Fig. 6B). The specificity of the enrichment of the CBD probe in leading edge filopodia in KO MEF was confirmed by showing that CBD-GFP, but not a mutant form lacking Cdc42 binding (CBD^H246,249D^; [55]), could enrich in filopodial tips (Fig. 6C). In order to further assess the relative protein and activation levels of Rac1 and Cdc42 in leading edge structures, we used the technique of Cho et al. [56] to isolate proteins from chemotactic cell projections found on the underside (i.e.- toward the chemoattractant) of 3 µm pores in Boyden chamber membranes. Fig. 6D shows that total Rac1 or Cdc42 protein levels did not differ in lysates of these projections. However, the level of activated Cdc42, based on binding to CBD-beads but not to mutantCBD-beads, was significantly higher in KO projection lysates than in those from WT cells. In contrast, GST-PAK-beads pulled down relatively equal levels of activated Rac1/Cdc42. Fig. 6E shows equal levels of purified CBD and PAK proteins used in overlay (Figs. 6A&C) and pulldown (Fig. 6D) assays.

These results suggest that selectively hyperactivated Cdc42 in KO cells causes a dominance of filopodia formation. This could be due to Rac activation or Cdc42 suppression by SSeCKS. To address this, KO MEF were transfected with either a dominant negative (DN) version of Cdc42 or a constitutively activated (CA) version of Rac, and then the cells were assessed for changes in chemotactic leading edge formations. Both DN-Cdc42 and CA-Rac1 expression caused the production of lamellipodial protrusions at the leading edge (Fig. 6F; 85% of transfected cells, based on EGFP co-expression, compared to vector-transfected cells), in contrast to the predominance of filopodia in vector-transfected KO MEF (>75% of transfected cells). The effect by DN-Cdc42 is consistent with a role for SSeCKS in inhibiting Cdc42 activation or localization of active Cdc42 to leading edge sites. However, we cannot exclude the possibility that SSeCKS is required for Rac1 activation given the ability of CA-Rac1 to induce lamellipodia in KO cells.

### Leading edge localization of the Cdc42-specific GEF, Frabin is sufficient to induce the morphological and hyper-chemotactic phenotype of SSeCKS-null cells

**Figure 7:**
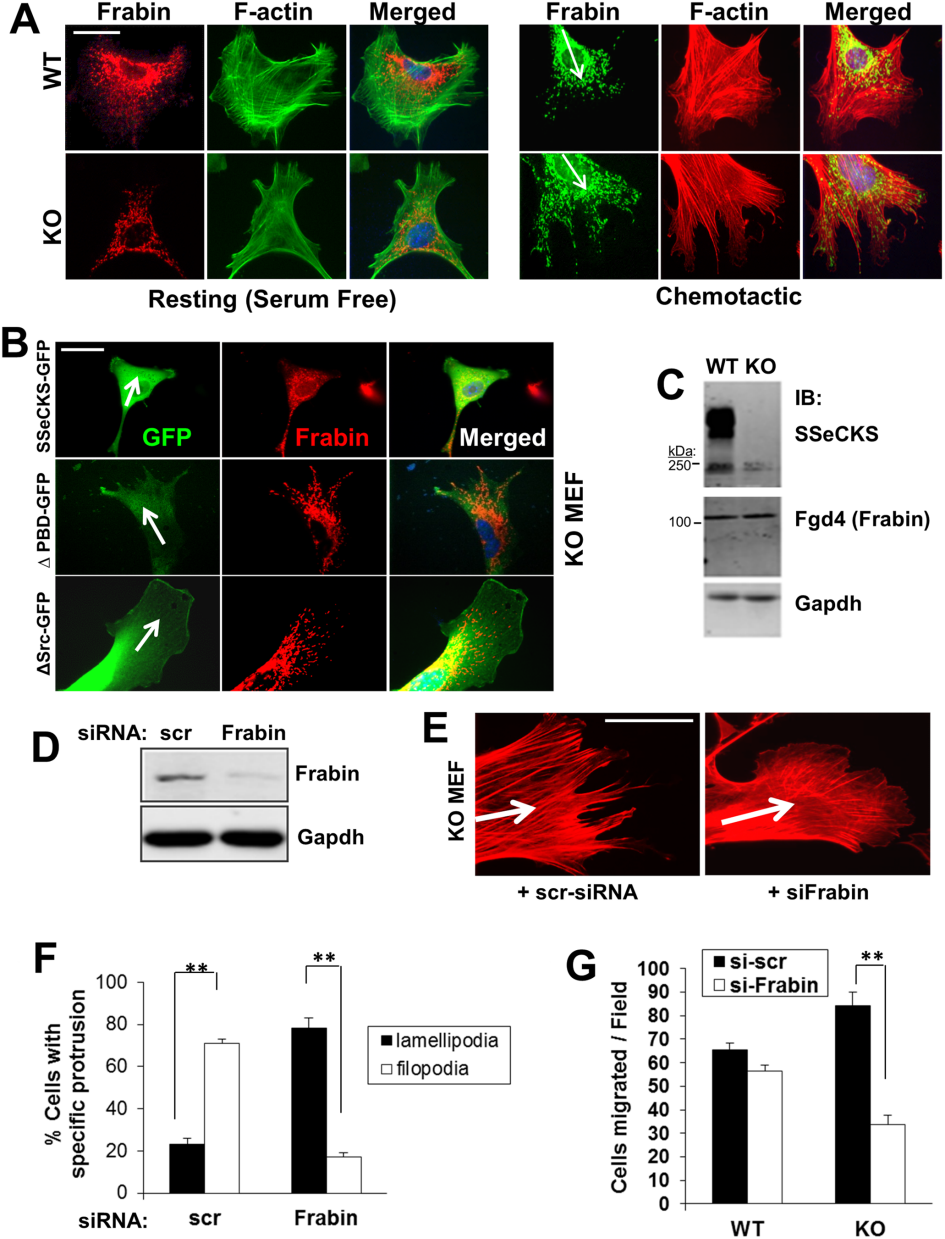
Enrichment of Frabin at the leading edge of KO MEF directs increased chemotaxis. *A*, IFA for Frabin or F-actin in resting vs. chemotactic (*arrows*) WT and KO MEF. *Scale bar,* 10 µm. *B*, IFA of GFP or Frabin in KO MEF re-expressing FL-, ΔPBD-, or ΔSrc-SSeCKS-GFP. *Scale bar,* 10 µm. *Arrow*, chemotaxis direction. *C*, IB analysis of SSeCKS, Frabin or Gapd levels in WT vs. KO MEF, relative to markers (*left*). *D,* IB analysis of Frabin or Gapdh protein levels in KO MEF cell lysates transfected with scrambled (scr) or Frabin-specific siRNA. *E*, IFA analysis of F-actin in KO MEF transfected with scr- or Frabin-siRNA. *Scale bar,* 5 µm. *Arrow*, chemotaxis direction. *F*, Quantification of KO MEF with leading edge lamellipodia vs. filopodia after transfection with scr- or Frabin-siRNA. *Error bars*, S.E. of 5 visual microscope fields with at least 10 cells/field in two independent experiments. **, p<0.005. *G*, Effect of scr- or Frabin-siRNA on WT or KO MEF chemotaxis. *Error bars*, S.E. of triplicates from two independent experiments. **, p<0.01.

We previously reported that although SSeCKS could attenuate v-Src-induced RhoA and Cdc42 activation, thereby inhibiting podosome/invadopodia formation, there was no interaction between these GTPases and SSeCKS based on co-immunoprecipitation [35]. The activation of Rho family GTPases is mediated by the translocation of GEFs to the membrane through their binding to PIPs [6]. We hypothesized that in the absence of SSeCKS, the localization of a Cdc42-specific GEF in filopodia structures at the leading edge, facilitated by the enrichment of PIP2 and PIP3, might drive the selective activation of Cdc42. Frabin (FGD1-related F-actin binding protein), a known Cdc42-specific GEF [57] that binds PIPs via two PH and one FYVE domain [58], can induce Cdc42 activation in the vicinity of actin structures leading to filopodia formation [59]. In the absence of a chemoattractant gradient, Frabin was distributed in a reticulate, cytoplasmic pattern with little association with plasma membrane structures in both WT and KO MEF (Fig. 7A; left panel). WT MEF exhibited increased levels of perinuclear Frabin compared to KO MEF. In the context of a chemoattractant gradient, however, Frabin enriched to the tips of filopodia in the leading edges of KO cells, whereas in WT MEF, it was relatively absent from the lamellipodia, and instead, concentrated in perinuclear regions (Fig. 7A, right panel). The ectopic expression of FL-SSeCKS in KO MEF restored leading edge lamellipodia formation, yet these structures were relatively devoid of Frabin staining (Fig. 7B). Similar results were obtained using an SSeCKS mutant, ΔSrc, deleted of its Src-binding domain (a.a. 153–166; ref. [27]). In contrast, ΔPBD-SSeCKS expression failed to induce lamellipodia, resulting in filopodia protrusions at the leading edge containing terminal enrichments of Frabin. Although SSeCKS affects the localization of Frabin, the level of total cellular Frabin (MWt. = 105 kDa) was not changed by SSeCKS (Fig. 7C). Importantly, we could not show Frabin-SSeCKS interactions in WT MEF lysates based on co-IP.

In order to address whether Frabin is required for the formation of filopodia protrusions found on KO chemotactic cells, we knocked down expression of Frabin with siRNA. Frabin siRNA reduced Frabin protein levels by 80% compared to cells transfected with control (scrambled) siRNA (Fig. 7D). Frabin deficiency resulted in the replacement of leading edge filopodia with lamellipodia (Figs. 7E&F) and with a stress fiber formation typical of WT cells (Fig. 7E). Importantly, knockdown of Frabin significantly inhibited chemotaxis of KO, but not WT cells (Fig. 7G). Taken together, these findings suggest that SSeCKS attenuates chemotaxis by preventing Frabin localization and activation at the leading edge, thereby attenuating the activation of Cdc42 and the growth of chemo-directional F-actin stress fibers.

### SSeCKS suppression of chemotaxis: mechanism

**Figure 8:**
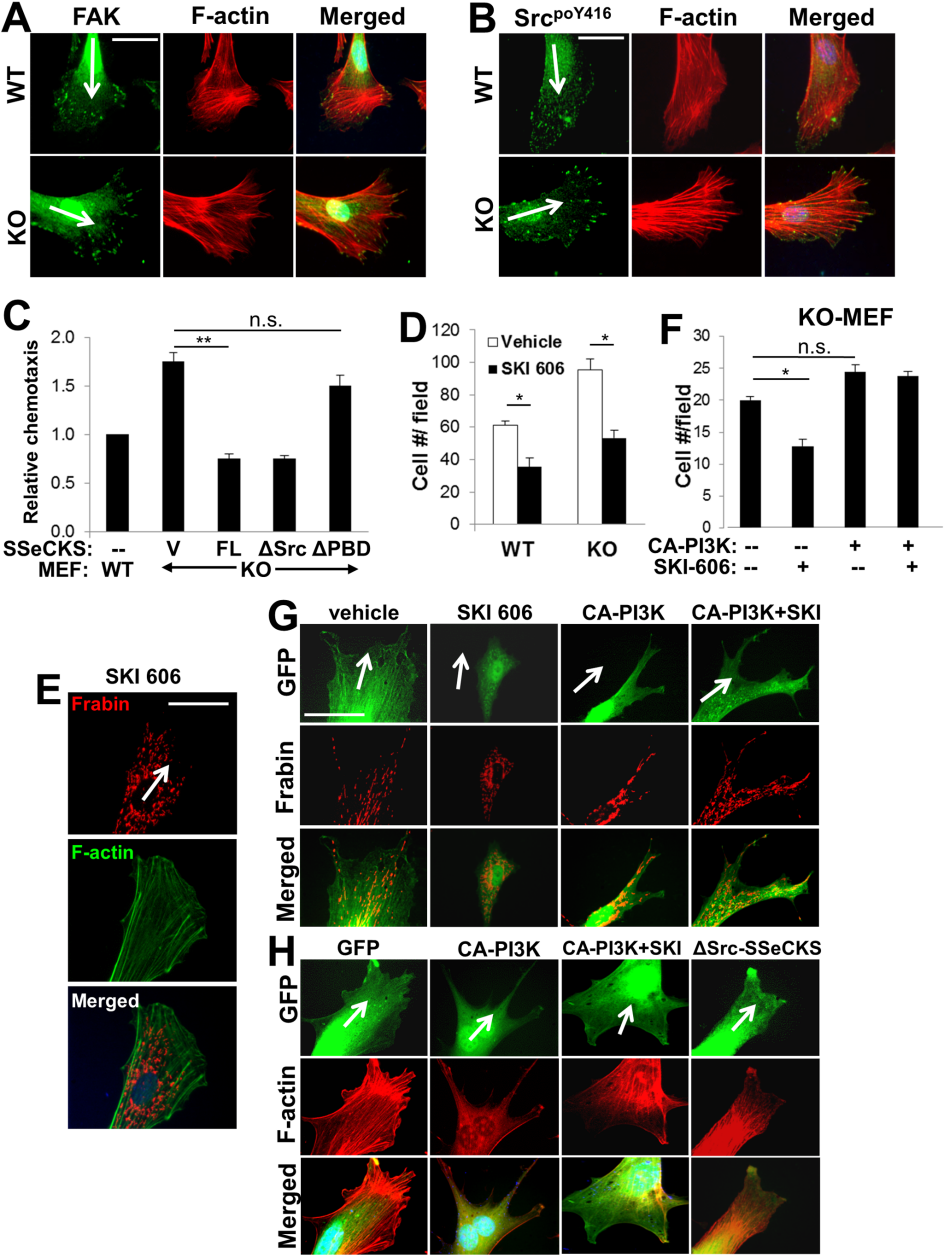
Enrichment of activated Src at the tips of leading edge filopodia in chemotactic KO MEF. IFA analysis of FAK and F-actin (*A*), or Src^poY416^ and F-actin (*B*) in chemotactic WT and KO MEF. Total levels of FAK or Src^poY416^ in the WT vs. KO MEF did not differ [28]. *Scale bar*, 10 µm. *Arrow*, chemotaxis direction. *C,* Relative chemotaxis of WT MEF or KO MEF transfected with vector (V), FL-, ΔPBD- or ΔSrc-SSeCKS-GFP. *Error bars*, S.E. of 5 visual microscope fields with at least 10 cells/field in two independent experiments. **, p<0.005; n.s., not significant. *D,* Chemotaxis (migrated cells/field) of WT cells treated with vehicle or SKI-606. *Error bars*, S.E. of 3 independent experiments. *, p<0.02. *E,* IFA analysis of Frabin and F-actin in KO MEF treated with SKI-606. *Scale bar*, 10 µm. Arrow, chemotaxis direction. *F,* Chemotaxis of KO MEF transfected with vector (–) or CA-PI3K and/or treated with SKI-606. *Error bars*, S.E. of 3 independent experiments. *, p<0.02; n.s., not significant. *G,* IFA analysis of GFP or Frabin in KO MEF transfected with pEGFP and CA-PI3K and then treated with vehicle or SKI-606. *Scale bar*, 10 µm. *Arrow*, chemotaxis direction. *H,* KO MEF transiently transfected with pEGFP alone or with plasmids encoding CA-PI3K or ΔSrc-SSeCKS, were treated with either vehicle or SKI-606 (1 µM) for 18 h, then subjected to directional chemotaxis assays, fixed and stained for F-actin. *Arrows*, chemotactic direction.

**Figure 9:**
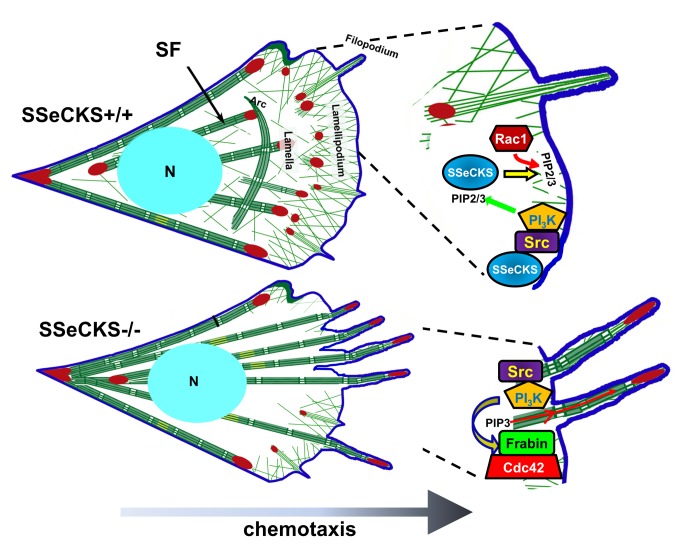
Model of chemotaxis leading edge structure/function by SSeCKS. In WT (SSeCKS+/+) cells (*top*), the ability of SSeCKS to scaffold Src at lipid raft sites allows for local activation of PI3K/AKT signaling, thereby producing locally enriched PIP2/3. SSeCKS’ additional ability to scaffold PIPs, including PIP2/3, at the chemotactic leading edge facilitates the local activation of Rac1, resulting in lamellipodial-dependent chemotaxis. In contrast, in the absence of SSeCKS’s scaffolding of PIPs, PIP3 enriches in budding filopodia, which attracts binding of Frabin through its PH and FYVE domains. This leads to local activation of Cdc42 and the subsequent domination of growing filopodia at the leading edge. Of note is that SSeCKS also affects actin cytoskeletal modeling not addressed directly in this paper, such as the generation of thickened, longitudinal stress fibers (SF) and the loss of arc SF in SSeCKS-null cells.

As mentioned above, SSeCKS encodes scaffolding domains for several possible mediators of cytoskeletal remodeling and leading edge formation, including domains for Src and PIP binding. Although the Src-scaffolding activity of SSeCKS was not necessary for the rescue of lamellipodia formation and Frabin translocation away from the leading edge (Fig. 7B), we noted that the leading edges of KO cells showed concentrations of FAK and active Src (Src^poY416^) at the tips of leading edge filopodia (Figs. 8A&B), although total Src protein and activation levels in MEF are not affected by SSeCKS [28]. Indeed, the FAK/Src configuration in KO MEF is consistent with increased enrichment of FAK and activated Src at the growing ends of so-called dorsal stress fibers, defined as being attached to focal adhesions at only one end [60]. Given that the enhanced chemotaxis of KO MEF requires PI3K activity (Fig. 3B), we endeavored a more comprehensive analysis of whether the enhanced chemotaxis and associated cytoskeletal and leading edge structures in KO cells is controlled locally by a Src-PI3K pathway that would directly influence production of local pools of PIP3 [12] and Frabin recruitment. Consistent with the Frabin localization and cytoskeletal remodeling result in Fig. 7B, FL and ΔSrc, but not ΔPBD-SSeCKS, could decrease the enhanced chemotaxis of KO MEF relative to levels in WT MEF (Fig. 8C). Inhibition of Src activity using the Src/Abl kinase inhibitor, SKI-606/bosutinib [61], decreased chemotaxis in WT and KO cells to statistically similar levels (Fig. 8D), yet induced lamellipodia formation and Frabin internalization from the leading edge in KO cells (Fig. 8E). However, although CA-PI3K did not increase chemotaxis (Fig. 8F), filopodia formation or Frabin enrichment at leading edge filopodia ends in KO cells (Fig. 8G), it did negate the ability of SKI-606 to inhibit chemotaxis (Fig. 8F) or to induce lamellipodia formation and Frabin internalization (Fig. 8G). This suggests that the enhanced chemotaxis of KO MEF is controlled by PI3K activity that is downstream of Src. Interestingly, whereas SKI-606 and ΔSrc-SSeCKS caused stress fibers to pull back from leading edge lamellipodia in KO cells (Figs. 8E&H), CA-PI3K seemed to induce fewer internal and more cell edge stress fibers (Fig. 8H), an effect that was not changed by SKI-606. Taken together with our earlier data, these findings suggest that in the absence of SSeCKS’ PIP scaffolding function, PIP2/3 concentrate at filopodial ends of the leading edge (Fig. 4). This leads to the enrichment of Frabin in these membrane sites via intrinsic PH and FYVE domains, and to the growing tips of F-actin fibers via an intrinsic FAB domain [62], resulting in increased chemotaxis through the local activation of Cdc42 (Fig. 9).

## Discussion

A fundamental component of cancer cell behavior is a dynamic change in cell motility driven by alterations in actin cytoskeletal remodeling. Indeed, cancer cells display increased parameters of chemotaxis and invasiveness, to the extent that these behaviors have been suggested as predictive biomarkers of metastasis [2]. The current study addresses the role of SSeCKS, a metastasis-suppressor, in regulating chemotaxis through the selective and spatial regulation at the leading edge of Rho family GTPases, GEFs, phosphoinositol phosphates and adhesion-regulating kinases such as FAK and Src.

SSeCKS suppresses chemotaxis in MEF and in cancer cells [26], but does not affect the cell motility measured in monolayer wound healing assays [36]. We noted that the leading edges of chemotactic SSeCKS-deficient cells were marked by accentuated filopodia-like protrusions, which indeed, are filopodia based on fascin staining, in contrast to the lamellipodia that typified the leading edges of SSeCKS-expressing cells. Additionally, the F-actin stress fibers in SSeCKS-deficient cells were thickened, highly polarized and directional towards the chemotactic gradient, and significantly, they reached to the tips of leading edge filopodia. In contrast, stress fibers in SSeCKS-expressing cells were thinner, and ended in angled bunches prior to the leading edge of lamellipodia. Our data suggest that the actin cytoskeletal remodeling mechanisms that SSeCKS suppresses results in decreased directional motility and velocity.

The ability of SSeCKS to attenuate chemotaxis is likely controlled by its direct scaffolding of phosphoinositol phosphates via three domains, PBD1-3. The PBD share homology, specifically a concentration of phenylalanine and lysine residues, with the so-called MARCKS membrane effector domain shown previously to bind various PIPs [63]. It is also likely that these domains, in conjunction with SSeCKS’ N-terminal myristylation, direct SSeCKS to specific plasma membrane regions temporally enriched with PIP2/3, such as the motile leading edge [64] or lipid rafts in non-motile cells [65]. Indeed, Yan *et al.* [22] showed that the PBD were sufficient to target human SSeCKS (AKAP12/Gravin) to membrane sites. Importantly, our data showed that the ability of SSeCKS to inhibit chemotaxis in MEF and MDA-MB-231 cells required the three PBD but not the Src scaffolding domain, strongly arguing that SSeCKS controls chemotaxis through competitive binding to or compartmentalization of PIPs. An argument against the former possibility is that SSeCKS levels do not correlate with changes in AKT protein or activation levels (Fig. 3C), which might be expected that if SSeCKS competed with the AKT-PH domain for PIP binding. However, our results are consistent with the latter possibility, namely that SSeCKS alters the level of active AKT enriching at the leading edge, most likely influenced by SSeCKS’ ability to attenuate the enrichment of PIP3, and to a lesser extent PIP2, in these protrusions.

The notion that SSeCKS regulates chemotaxis through the physical scaffolding and organization of PIPs (Fig. 9), and the subsequent selective enrichment of signaling proteins at the leading edge was strengthened by our data showing increased levels of AKT, PKC-ζ and Cdc42 at the leading edge of KO vs. WT MEF, although total cellular levels of these proteins were unaffected by SSeCKS. The exception was the polarity protein, Par6, normally recruited to the leading edge by activated Cdc42, which was not enriched in KO cell leading edges. Indeed, SSeCKS directly scaffolds PKC-ζ, as it does for other PKC isoform classes [29], suggesting a direct mechanism for affecting localization. It is noteworthy that we could not show co-IP of AKT or Cdc42 with SSeCKS suggesting that SSeCKS affects their membrane localization indirectly. These recruitments correlated with increased levels of activated Cdc42 (GTP-bound) in KO cell leading edges as assessed by overlay assays with epitope-tagged WASP-CBD domains and by purification of chemosensing pseudopodia. In contrast, KO and WT leading edges showed no difference in the binding of epitope-tagged PAK-PBD, which recognizes both activated Rac and Cdc42 [effector binding domains specific for activated Rac alone have not been identified]. Taken together with our findings that KO cell leading edges have relatively decreased levels of total Rac1 yet increased levels of activated Cdc42, it is likely that our PAK-PBD result suggests that SSeCKS normally facilitates localization of Rac1 to activation sites on the motile leading edge. Consistent with this, the overexpression of CA-Rac1 was sufficient to induce leading edge lamellipodia in KO MEF, as was the overexpression of DN-Cdc42.

Our data demonstrated that the Cdc42-specific GEF, Frabin, was selectively enriched at the tips of filopodia-like protrusions at the leading edge of KO MEF, compared with little or no enrichment at leading edge lamellipodia in WT cells (Fig. 9). This enrichment is likely facilitated by multiple PIP-binding domains encoded by Frabin. Indeed, re-expression of FL- or ΔSrc-SSeCKS, but not ΔPBD-SSeCKS, induced lamellipodial protrusions at the leading edge which were relatively devoid of Frabin enrichment. Conversely, the siRNA-mediated knockdown of Frabin suppressed the formation of leading edge filopodia and enhanced chemotaxis in KO MEF. This argues that in the absence of SSeCKS, Frabin is responsible for the selective activation of Cdc42 at leading edges, resulting in the formation of filopodia during chemotaxis [45]. Why the SSeCKS-Frabin-Cdc42 axis controls single-cell chemotaxis vs. monolayer wound healing motility dynamics is unclear. However, it is possible that wound healing motility is less dependent on this control axis because it reflects collective migration driven by changes in adhesion tension at each cell’s leading edge. In contrast, chemotaxis is driven by an enrichment of chemoattractant receptors (e.g.- PDGFR) at the leading edge. Thus, scaffolding proteins, such as SSeCKS, that can locally regulate Src/PI3K/AKT signaling, PIP generation and Rac1/Cdc42 activation/localization (Fig. 9), might play critical roles in regulating chemotaxis vs. collective migration.

Although SSeCKS can control adhesion- and growth factor- signaling by directly scaffolding pools of active Src away from FAK complexes [27], this activity was not required for SSeCKS’ ability to rescue lamellipodia formation and chemotaxis inhibition. This notion that Src plays a generic role (i.e.- not involved in SSeCKS’ chemotaxis control) is borne out by our data that Src kinase inhibition suppressed WT and KO cell chemotaxis equally. However, PI3K, and its ability to generate PIP3, is likely downstream of Src in our system because CA-PI3K could negate the ability of SKI-606 to suppress chemotaxis and Frabin localization at leading edge filopodia in KO cells. Additionally, CA-PI3K increased chemotaxis equally in WT or KO cells, although it was not sufficient to induce any of the leading edge phenotypes found in KO cells. Therefore, it is likely that SSeCKS controls chemotaxis through a spatial scaffolding of PIPs, resulting in the recruitment of signaling mediators and cytoskeletal architecture that favors Cdc42-induced filopodia formation and directional motility. If this putative scaffolding function plays a role in SSeCKS’ ability to suppress metastasis, this implies that the loss of SSeCKS in advanced cancer [19] facilitates metastatic cell motility parameters (e.g.- directional chemotaxis and invasiveness) through the selective activation of Cdc42 and its cytoskeletal remodeling pathways.

## Supporting Information

Checklist S1 **NC3Rs ARRIVE Guidelines Checklist.**
(PDF)
